# The Viral Chemokine MCK-2 of Murine Cytomegalovirus Promotes Infection as Part of a gH/gL/MCK-2 Complex

**DOI:** 10.1371/journal.ppat.1003493

**Published:** 2013-07-25

**Authors:** Felicia M. Wagner, Ilija Brizic, Adrian Prager, Tihana Trsan, Maja Arapovic, Niels A. W. Lemmermann, Jürgen Podlech, Matthias J. Reddehase, Frederic Lemnitzer, Jens Bernhard Bosse, Martina Gimpfl, Lisa Marcinowski, Margaret MacDonald, Heiko Adler, Ulrich H. Koszinowski, Barbara Adler

**Affiliations:** 1 Max von Pettenkofer-Institute for Virology, Ludwig-Maximilians-University Munich, Munich, Germany; 2 Department of Histology and Embryology, Faculty of Medicine, University of Rijeka, Rijeka, Croatia; 3 Institute for Virology and Research Center for Immunology (FZI), University Medical Center of the Johannes Gutenberg-University Mainz, Mainz, Germany; 4 Laboratory of Virology and Infectious Disease, Rockefeller University, New York, New York, United States of America; 5 Research Unit Gene Vectors, German Research Center for Environmental Health (GmbH), Munich, Germany; Oregon Health and Science University, United States of America

## Abstract

Human cytomegalovirus (HCMV) forms two gH/gL glycoprotein complexes, gH/gL/gO and gH/gL/pUL(128,130,131A), which determine the tropism, the entry pathways and the mode of spread of the virus. For murine cytomegalovirus (MCMV), which serves as a model for HCMV, a gH/gL/gO complex functionally homologous to the HCMV gH/gL/gO complex has been described. Knock-out of MCMV gO does impair, but not abolish, virus spread indicating that also MCMV might form an alternative gH/gL complex. Here, we show that the MCMV CC chemokine MCK-2 forms a complex with the glycoprotein gH, a complex which is incorporated into the virion. We could additionally show that mutants lacking both, gO and MCK-2 are not able to produce infectious virus. Trans-complementation of these double mutants with either gO or MCK-2 showed that both proteins can promote infection of host cells, although through different entry pathways. MCK-2 has been extensively studied in vivo by others. It has been shown to be involved in attracting cells for virus dissemination and in regulating antiviral host responses. We now show that MCK-2, by forming a complex with gH, strongly promotes infection of macrophages in vitro and in vivo. Thus, MCK-2 may play a dual role in MCMV infection, as a chemokine regulating the host response and attracting specific target cells and as part of a glycoprotein complex promoting entry into cells crucial for virus dissemination.

## Author Summary

Several human herpesviruses form alternative gH/gL complexes which determine the tropism for different cell types. For murine cytomegalovirus (MCMV), a gH/gL/gO complex has recently been characterized. Here, we present the identification and characterization of an alternative gH/gL/MCK-2 complex which promotes MCMV spread and is important for efficient infection of macrophages in vitro and in vivo. Association of the MCMV CC chemokine MCK-2 with a glycoprotein complex promoting virus entry is a novel function for the well-characterized MCK-2. Virus mutants lacking MCK-2 have been shown to exhibit a reduced capacity to attract leukocytes and a disregulated T cell control of the MCMV infection in vivo. These defects can be attributed to the chemokine function of MCK-2. Yet, the observation that MCK-2 knock-out mutants additionally are impaired in infecting leukocytes in vivo is consistent with our new finding that MCK-2 forms a glycoprotein complex promoting entry into monocytic cells. gH/gL complexes associating with multifunctional proteins add a new level of complexity to the interpretation of infection phenotypes of the respective knock-out herpesviruses.

## Introduction

Herpesviruses enter their host cells either by fusion of the viral envelope with the plasma membrane or with membranes of endocytotic vesicles. The fusion process is promoted by a concerted action of the conserved viral glycoproteins gB, gH, and gL [1] of which gH and gL consistently form a tight heterodimer [2], [3]. These three glycoproteins can promote receptor recognition and subsequent fusion as has been shown for the entry of Epstein-Barr virus (EBV) into epithelial cells [1]. Often, gB and gH/gL are not sufficient to promote receptor recognition. For instance, entry may depend on further envelope glycoproteins, as has been shown for gD of Herpes simplex virus [1], or on gH/gL forming tight complexes with additional viral proteins, as has for example been shown for the gH/gL/gp42 complex of EBV [4]–[6], the gH/gL/Q1/Q2 complex of HHV6 [7], or the gH/gL/pUL(128,130,131A) complex of HCMV [8]–[10].

For HCMV, two gH/gL complexes have been identified. In vitro, formation of gH/gL/gO ensures efficient production of infectious supernatant virus and promotes entry into a restricted set of cells by fusion at the plasma membrane [11], [12]. In the absence of gO, HCMV spreads in a cell-associated manner [11]. A restriction of cell tropism for mutants lacking gO has not been observed. The second complex, gH/gL/pUL(128,130,131A) promotes entry into a broad range of HCMV host cells including endothelial, epithelial, and dendritic cells [13], [14] by using endocytotic pathways [15]–[17]. Data published recently strongly suggest that gH/gL/gO and gH/gL/pUL(128,130,131A) promote virus entry through distinct cellular receptors [18], [19]. Depending on the HCMV strain analyzed, gO has been found to be incorporated into the virion or not [20]–[22]. The UL128, UL130 and UL131A gene products have consistently been shown to be incorporated into the virion [8], [9], [20], [21], [23], [24]. Their precise functions in the entry process have not yet been determined. It is also not known what the exact functions of gH/gL/gO and gH/gL/pUL(128,130,131A) are in the infection of humans. In a recent publication, we could show that the gH/gL complexes of HCMV are distributed to distinct virus populations which consequently differ in their cell tropism. In vitro, host cells like fibroblasts and endothelial cells either released or retained the population promoting infection of endothelial cells. We have proposed that, by determining the target cells of their virus progeny, host cells may route infection in vivo [23].

Infection of mice with MCMV serves as an animal model for the HCMV infection. We have recently identified the m74 ORF of MCMV as a functional homolog of HCMV gO [15]. Although the MCMV genome does not contain sequence homologs for HCMV UL128, UL130, and UL131A, the relative positions of the MCMV m130, m131/129, and m133 ORFs within the MCMV genome are comparable to the positions of the UL128, UL130, and UL131A ORFs in the HCMV genome. The m130 gene product has not been characterized. Deletion of m133 has been shown to result in reduced virus growth in salivary glands in vivo [25], [26]. The ORFs of m131 and m129 are fused by a splicing event which results in a protein product designated MCK-2 [27], [28]. The m131-derived part of MCK-2 contains, like the UL128 protein of HCMV, a CC (ß) chemokine domain. Besides that, MCK-2 does not show further sequence homologies to the UL128 gene product. MCK-2 and synthetic peptides of the m131 ORF or the complete MCK-2 have been shown to attract monocytes confirming its predicted chemokine activity [29], [30]. When mice are infected with MCMV mutants lacking MCK-2 the most apparent phenotype is a reduced virus production in salivary glands [28], [31], [32]. MCK-2 knock-out mutants are impaired in recruiting leukocytes which might serve as vehicles for virus dissemination [29], [30], [32]. Some populations of the attracted leukocytes have been shown to control virus specific CD8^+^ T cell immunity [33]. Yet, these populations differ from the myelomonocytic cells which are infected at the site of virus entry [30], [33]. Notably, MCK-2 knock-out viruses have additionally been shown to exhibit a 10-fold lower capacity to infect attracted myelomonocytic leukocytes [30].

Here, we report a completely new role for MCK-2, namely, as part of a gH/gL/MCK-2 complex promoting entry into macrophages. This offers an explanation for the hitherto unexplained low infection capacities of MCK-2 knock-out viruses for leukocytes in vivo [30]. The gH/gL/MCK-2 complex can complement the function of the gH/gL/gO complex of MCMV with respect to virus spread in vitro and strongly increases the efficiency of MCMV in infecting macrophages in vitro and in vivo. We propose that MCK-2 might have a dual role in infection, one as a chemokine attracting cells regulating the host immune response or attracting MCMV target cells and one in infecting viral target cells promoting subsequent virus dissemination.

## Results

### MCMV MCK-2 forms a complex with MCMV glycoprotein gH in virions

HCMV and MCMV lacking gO both show the same spread phenotype in vitro, namely, strongly reduced titers of infectious virus in supernatants of infected cells and a focal spread pattern. For HCMV, we could show that the residual focal spread of mutants lacking gO is dependent on the alternative gH/gL/pUL(128,130,131A) complex [15]. To find out whether MCMV also forms an alternative gH/gL complex, we infected cells with bacterial artificial chromosome (BAC)-derived wildtype MCMV and precipitated gH-associated proteins from extracts of virus released into the supernatant by using an antibody specific for MCMV gH [34]. The precipitates were separated on SDS-polyacrylamide gels, proteins extracted from gel slices and then analyzed by liquid chromatography-tandem mass spectrometry. The obtained peptides were compared to MCMV gene translations. One prominent hit was a LLCLVR peptide which matches the C-terminus of the m131 ORF which together with the m129 ORF forms the MCMV MCK-2 protein (data not shown).

**Figure 1:**
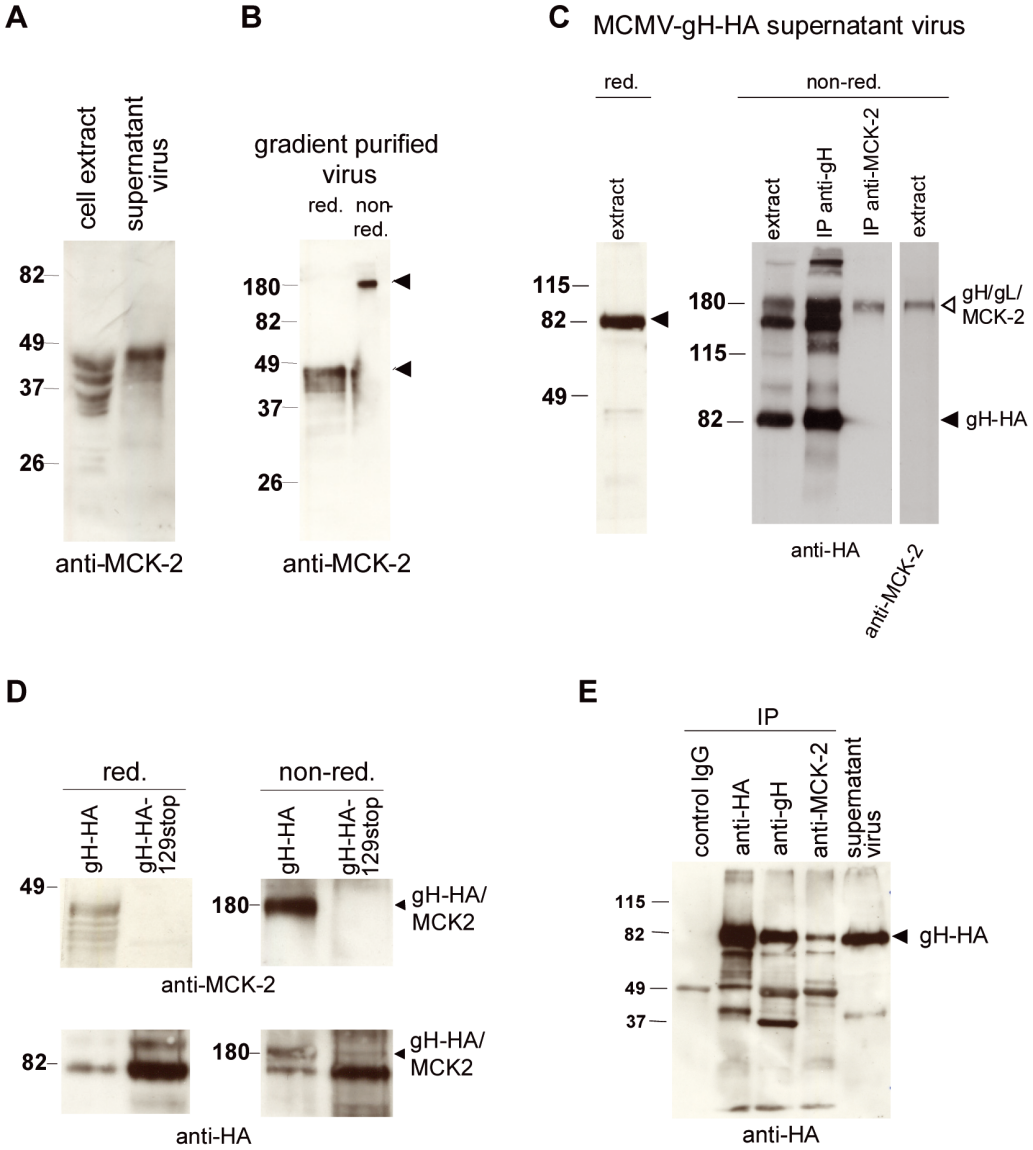
MCK-2 is a virion protein forming a high-molecular weight complex co-migrating with gH. (A–E) Lysates of cells, supernatant virus, or precipitated proteins were separated on SDS-polyacrylamide gels and analyzed by Western blot using the anti-MCK-2 antibody 5A5 to detect MCK-2 or the anti-HA antibody 3F10 to detect gH-HA. The positions of the molecular weight markers (kDa) are indicated. (A) Infected cells and supernatant virus from NIH3T3 cells infected with wildtype MCMV were harvested 5 days after infection and lysed in reducing sample buffer. (B) Gradient purified wildtype MCMV was lysed in reducing (red.) and non-red. sample buffer. The MCK-2 specific bands are indicated by arrows. (C) Supernatant virus from NIH3T3 cells infected with MCMV-gH-HA was either directly lysed in red. or non-red. sample buffer (extract) or proteins were precipitated from lysates with anti-gH or anti-MCK2 antibodies and then analyzed under non-red. conditions. Monomeric gH-HA (black arrow) and gH/gL/MCK-2 (white arrow) are indicated by arrows. (D) Supernatant virus from NIH3T3 cells infected with MCMV-gH-HA or MCMV-gH-HA/129stop was lysed in red. or non-red. sample buffer. The upper panel shows extracts prepared under red. and non-red. conditions and stained with an anti-MCK-2 antibody. The lower panel shows the same extracts stained with an anti-HA antibody to detect gH-HA. The gH/MCK-2 band formed under non-red. conditions is indicated by an arrow. The MCMV-gH-HA/129stop extracts showed an at least fivefold higher protein content. (E) NIH3T3 cells were infected with MCMV-gH-HA and supernatant virus harvested 5 days after infection. Lysates of supernatant virus were either directly analyzed for gH-HA expression (supernatant virus) or after immunoprecipitation using a mouse IgG control antibody, an anti-HA antibody, an anti-gH antibody, or an anti-MCK2 (2H9) antibody recognizing the m131 region of MCK-2. The gH-HA specific protein band is indicated.

On Western blots, MCK-2 appears as multiple glycosylated forms running between 30 and 45 kDa ([27] and (Fig. 1A)). When we prepared extracts of supernatant virus, MCK-2 ran at a slightly higher molecular weight than MCK-2 from extracts of infected cells (Fig. 1A). This points towards a differentially modified protein. A similar pattern has been shown for MCK-2 secreted from transfected cells [27]. MCK-2 could also be detected in extracts of gradient purified virus which strongly suggests that it is incorporated into virions (Fig. 1B). Under non-reducing conditions, MCK-2 migrated at a molecular weight of about 180 kDa (Fig. 1B), which argues for MCK-2 forming a tight complex with other viral proteins in virions. As there is no antibody available which recognizes MCMV gH in Western blots, we constructed an MCMV BAC which expresses a C-terminally HA-tagged gH (Fig. S1) which grew like wildtype virus (Fig. S2). gH-HA could easily be detected in extracts of supernatant virus (Fig. 1C). Under reducing conditions it migrated at the expected molecular weight of about 85 kDa [34]. When supernatant virus from cells infected with MCMV-gH-HA was analyzed under non-reducing conditions, two prominent high molecular weight bands, one running slightly above and one running below the 180 kDa marker could be detected for gH-HA. The upper band co-migrated with MCK-2 (Fig. 1C). Whereas an anti-gH antibody could precipitate all gH bands visible in extracts of supernatant virus, an anti-MCK-2 antibody specifically precipitated the band co-migrating with MCK-2 (Fig. 1C). This band very likely represents a gH/gL/MCK-2 complex. The prominent gH-HA positive band below 180 kDa could represent a gH/gL complex consisting of gH-HA and the 274 amino acid long gL. We could also show that the upper band represents a complex containing gH and MCK-2 by using an MCMV-gH-HA mutant which carries a disrupted MCK-2 ORF (MCMV-gH-HA/129stop) (Fig. S1). This mutant does not express MCK-2 (Fig. 1D, upper panel) and lacks the upper gH-HA band under non-reducing conditions (Fig. 1D, right lower panel). The protein extracts of the gH-HA/m129stop mutant were at least five times more concentrated than the extracts of the gH-HA virus which can be seen from the strength of the gH-HA band under reducing conditions and the lower gH-HA band under non-reducing conditions (Fig. 1D, lower panel). Thus, it could be excluded that the gH/MCK-2 band had escaped detection. To show that the anti-MCK-2 antibody specifically co-precipitates gH and to confirm the co-precipitation of MCK-2 which we had found by mass spectrometry analysis of proteins precipitated with an anti-gH antibody, we performed the reverse co-immunoprecipitation using an anti-MCK-2 antibody to precipitate MCK-2 associated proteins. An extract was prepared from supernatant virus of cells infected with MCMV-gH-HA and aliquoted. Anti-gH and anti-HA antibodies readily precipitated gH-HA from this extract (Fig. 1E). The anti-MCK-2 antibody clearly co-precipitated gH-HA, whereas a mouse control IgG antibody did not (Fig. 1E).

**Figure 2:**
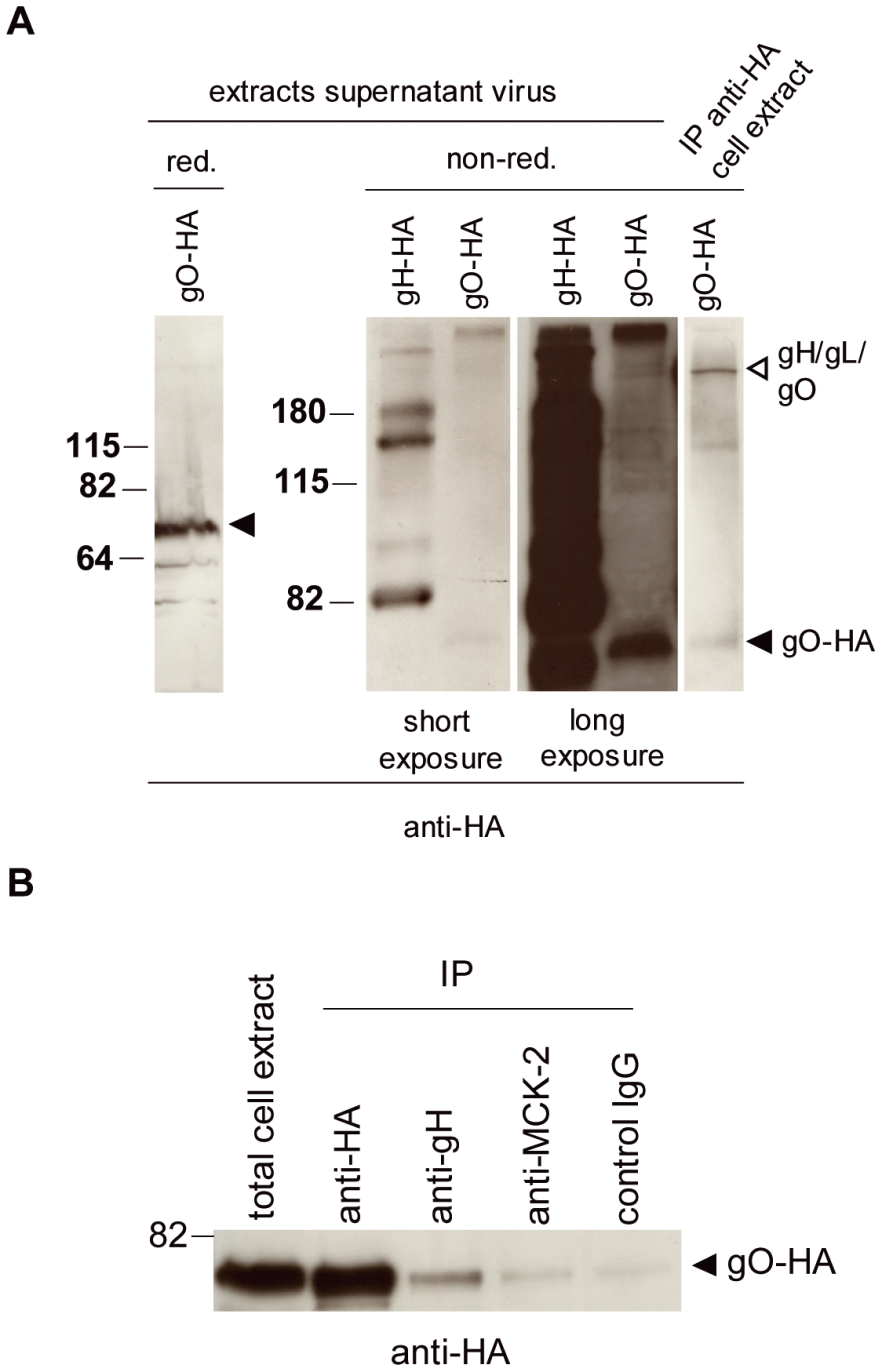
MCK-2 associates with gH in a complex distinct from gH/gL/gO. (A) Supernatant virus from NIH3T3 cells infected with MCMV-gH-HA or MCMV-gO-HA was lysed in red. or non-red. sample buffer. gO-HA and gH-HA were detected with an anti-HA antibody. Monomeric gO-HA (black arrow) and gH/gL/gO (white arrow) are indicated by arrows. Two exposures of the Western blots showing non-red. extracts are depicted. As a control, gO-HA was precipitated from a lysate of MCMV-gO-HA infected cells with an anti-HA antibody and analyzed under non-red. conditions. (B) MEF were infected with MCMV-gO-HA, and three days after infection, cells were harvested and either directly analyzed for gO-HA expression (total cell extract) or after immunoprecipitation using an anti-HA antibody, an anti-gH antibody, an anti-MCK2 (2H9), or a mouse IgG control antibody. (A–B) The gO-HA specific protein band runs at about 70 kDa. The positions of the molecular weight markers (kDa) are indicated.

To show that gH/gL/gO and gH/gL/MCK-2 are alternative complexes, we used an MCMV BAC expressing HA-tagged gO. This BAC was generated by adding an HA-tag to the 3′ end of the m74 ORF and by introducing a duplication of the overlapping C-terminus of the m73 ORF (gN) to preserve the function of gN (gO-HA, Fig. S1). MCMV-gO-HA grew like wildtype virus in fibroblasts (data not shown). We have previously shown that HA-tagged gO expressed in the virus context forms a complex of more than 200 kDa which can be precipitated with anti-gH and anti-HA antibodies [15]. To show that this complex is different from the complex formed by gH and MCK-2, we compared extracts from supernatant virus from MCMV-gH-HA and MCMV-gO-HA. gO-HA could easily be detected in extracts of supernatant virus (Fig. 2A). Under reducing conditions it migrated at the expected molecular weight of about 70 kDa [15]. Under non-reducing conditions a weak band representing the gH/gL/gO complex could only be detected after a very long exposure of the Western blots (Fig. 2A). In Figure 2A extracts of supernatant virus from cells infected with either MCMV-gH-HA or MCMV-gO-HA are depicted side by side and stained for the HA-tag. To show the position of the gH/gL/gO complex more clearly, an anti-HA immunoprecipitation from extracts of infected cells was included [15]. The comparison of extracts from MCMV-gO-HA and MCMV-gH-HA shows that the gO and MCK-2 complexes clearly have different molecular weights. Additionally, anti-MCK-2 which had co-precipitated gH from lysates of supernatant virus (Fig. 1 E), did not co-precipitate gO-HA from extraxts of infected cells, whereas anti-gH antibodies clearly co-precipitated gO-HA (Fig. 2B). These findings strongly support that gH/gL/gO and gH/gL/MCK-2 are indeed distinct complexes. In total cell extracts (data not shown) and in extracts of supernatant virus infected with the gH-HA virus, the anti-HA antibody could not detect a complex corresponding to the gH/gL/gO complex (Fig. 1C and Fig. 2A), a failure which might be due to a loss of the accessibility of the HA-tag of gH when the gH/gL/gO complex is formed.

### MCMV lacking MCK-2 shows a reduced capacity to infect macrophages

**Figure 3:**
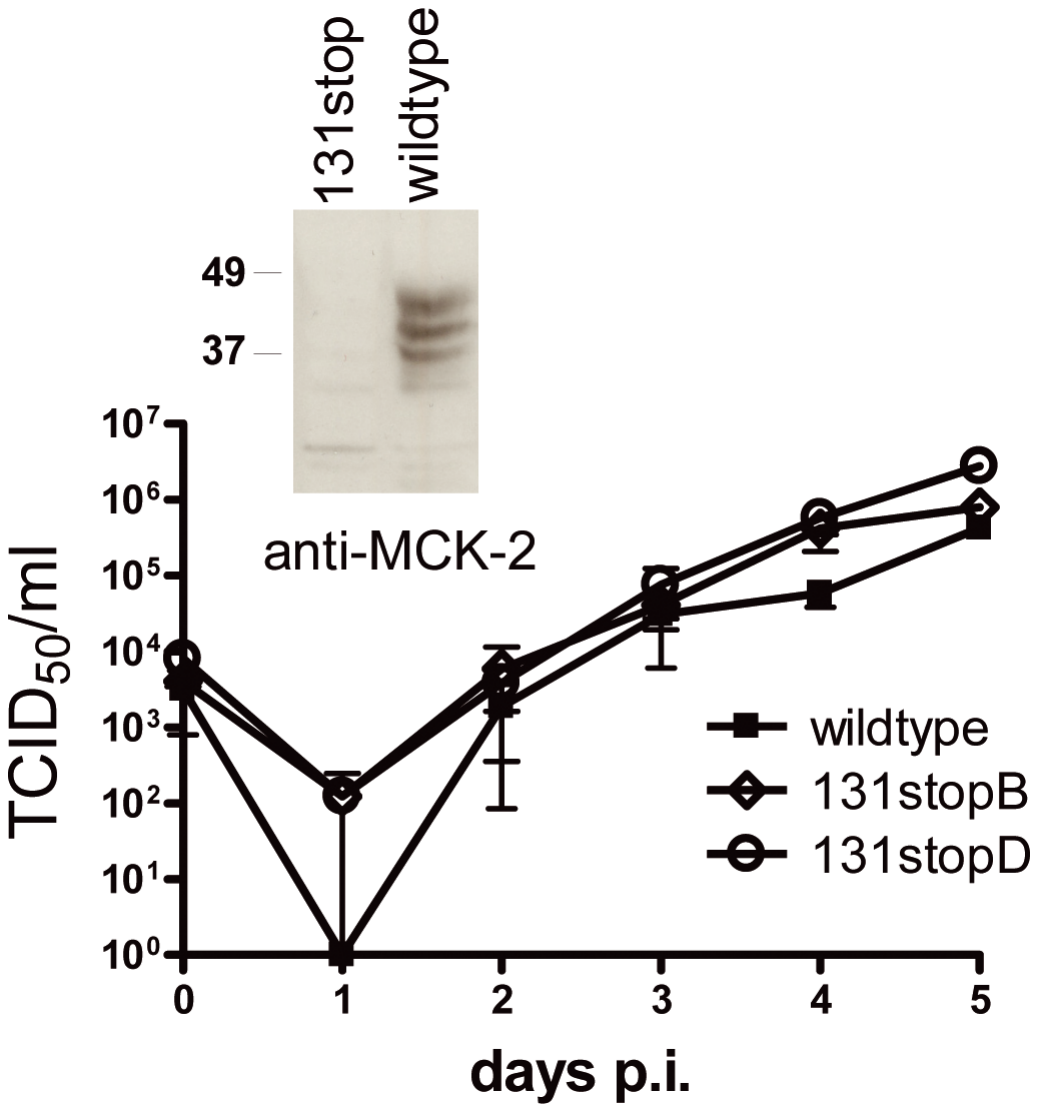
Multistep growth curves in NIH3T3 cells infected with wildtype MCMV and 131stopB and 131stopD mutants. Cells were infected at an m.o.i. of 0.1, supernatants were harvested every 24 hours and titrated. Shown are means +/− SD of three independent growth curves for each virus. The insert depicts the loss of MCK-2 by staining extracts of NIH3T3 cells infected with wildtype or 131stopB virus with an anti-MCK-2 (5A5) antibody. p.i., post infection.

To analyze the role of MCK-2 within a glycoprotein complex promoting entry, we constructed BAC-derived MCMV mutants in which the m131/129 reading frame was disrupted by stop cassettes and which do not express MCK-2 (Fig. S1). MCK-2 knock-out mutants previously constructed by others have been extensively studied in vivo and shown to exhibit defects in recruitment of leukocytes, in virus dissemination, and in growth in salivary glands [28], [30], [32]. We have recently shown that an MCK-2 mutant of the MCMV strain Smith which was cloned as a BAC also showed a reduced growth in salivary glands in vivo [31]. None of these mutants has been reported to show attenuation in vitro [28], [29], [31]. We could confirm this for two clones of the 131stop mutant (Fig. 3). Multistep growth curves even exhibited a marginal growth advantage for the MCK-2 knock-out mutants at days 4 and 5 after infection, yet, even three independent growth curves could not show that these differences were statistically significant.

**Figure 4:**
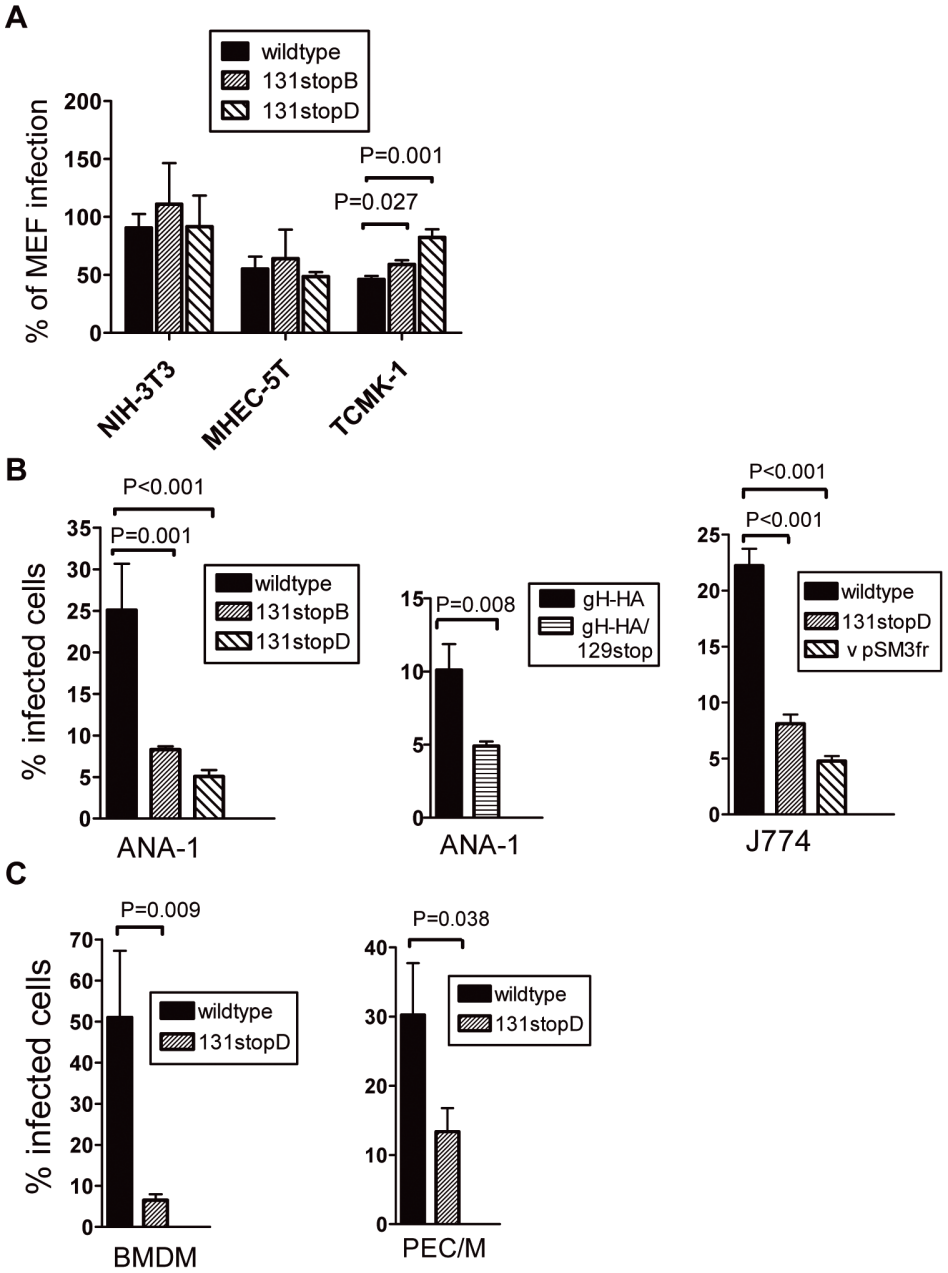
Expression of full-length MCK-2 facilitates the infection of macrophages in vitro. (A) MEF, NIH3T3, MHEC-5T and TCMK-1 cells were plated in 96 well plates, infected with wildtype MCMV and 131stop mutants of MCMV at an m.o.i. of 0.5, and 6 h after infection stained for MCMV immediate early 1 (IE1) protein by indirect immunofluorescence. Each infection was done in triplicates and for each well IE1^+^ cells were counted and the means of these counts related to the mean of IE1^+^ MEF. The value for MEF was set to 100%. Shown are means +/− SEM of at least 4 independent experiments. Infection of TCMK-1 with m131stop mutants was significantly enhanced compared to wildtype infection. The P values (Student's t-test) are indicated in the histograms. (B) Immortalized macrophages ANA-1 (left and middle panel) and J774 (right panel) were infected in suspension with wildtype MCMV or MCMV-gH-HA carrying a wildtype MCK-2 or with the MCK-2 mutants m131stopB or D, MCMV-gH-HA/129stop, or pSM3fr BAC-derived virus. The numbers of infected macrophages were determined by intracellular FACS staining for IE1^+^ cells. All infections with MCK-2 mutants showed significantly lower numbers of infected macrophages than infections with wildtype virus. The P values (Student's t-test) are indicated in the histograms. Shown are means +/−SEM of 3 to 4 independent experiments. (C) Primary BMDM (left panel) and cells in the macrophage-enriched gate of PEC (PEC/M) (right panel) were infected with wildtype virus or the MCK-2 mutant 131stopD as described under (B). The numbers of infected cells were significantly lower for the 131stop mutant. The P values (Student's t-test) are indicated in the histograms. Shown are means +/−SEM of 3 independent experiments. (B and C) All virus preparations have been titrated on MEF and for each experiment MEF were infected in parallel to confirm that wildtype and mutant viruses infected comparable percentages of MEF.

For HCMV, it has been shown that the inability to form the alternative gH/gL/pUL(128,130,131A) complex abolishes the tropism for cells like endothelial and epithelial cells. Thus, we infected primary (MEF) and immortalized fibroblasts (NIH3T3), endothelial cells (MHEC-5T), and epithelial cells (TCMK-1) with wildtype virus and 131stop mutants and compared the infection capacities by staining the cells for expression of the immediate early 1 (IE1) protein of MCMV. The numbers of infected MEF cells were set to 100% and numbers of infected NIH3T3, MHEC-5T, and TCMK-1 were expressed as percent of MEF infection (Fig. 4A). No significant differences in infection capacities for fibroblasts or endothelial cells could be detected when wildtype and 131stop MCMV were compared. Only infection of TCMK-1 epithelial cells was slightly but significantly enhanced. As staining for expression of IE1 reflects successful entry, but not the ability to replicate in certain cell types, we also tested virus production of wildtype and 131stop viruses in these cell types but could not detect any differences (data not shown).

When MCK-2 knock-out mutants were analyzed in vivo, reduced capacities to infect myelomonocytic leukocytes were observed when compared to wildtype infections [30]. To find out whether this is also observed when closely related cells like macrophages are infected in vitro, we infected macrophage cell lines like ANA-1 or J774, primary bone marrow derived macrophages (BMDM), or macrophages directly from peritoneal exudates (PEC/M). For the latter, infection was studied for cells in the macrophage-enriched gate of peritoneal exudate cells (PEC) from untreated BALB/c mice ([35] and Fig. S3). ANA-1 cells were infected with wildtype virus, two clones of the 131stop mutant, and a gH-HA/129stop mutant (Fig. 4B). J774 cells were infected with wildtype virus, a 131stop mutant, and a pSM3fr BAC-derived virus which carries a stop mutation in m129 and shows the typical reduction of growth in salivary glands after in vivo infection [31] (Fig. 4B). BMDM and PEC were infected with wildtype virus and a 131stop mutant (Fig. 4C). For all macrophages tested, all mutants unable to express an intact MCK-2 showed a strongly and significantly reduced capacity to infect macrophages (Fig. 4B and C). To exclude that the differences in infection capacities are due to soluble MCK-2 produced by cells infected with wildtype virus and not to the presence of a gH/gL/MCK-2 complex promoting infection, virus pelleted from supernatants of infected cells and purified by centrifugation through sucrose cushions was used.

**Figure 5:**
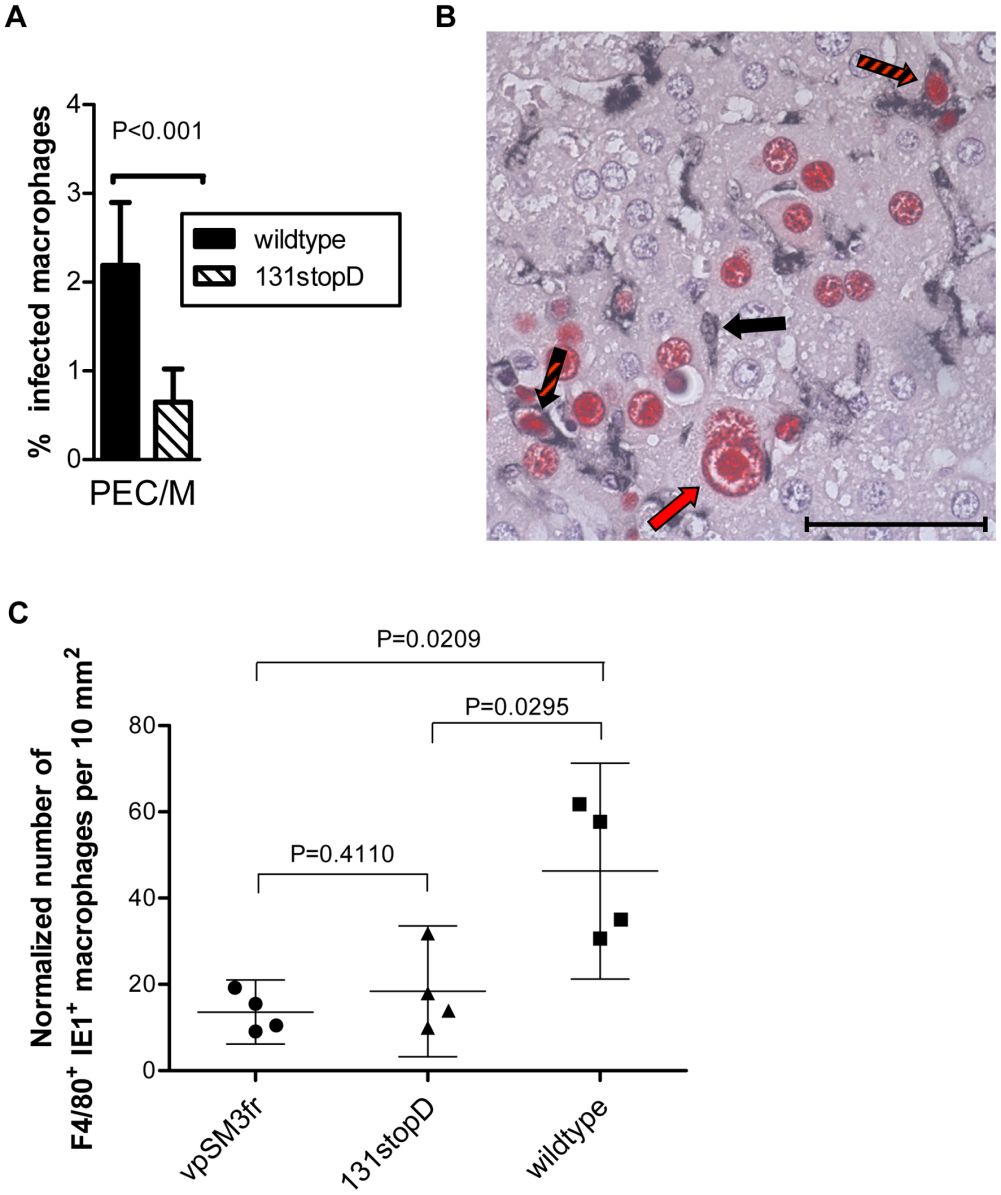
Expression of full-length MCK-2 facilitates the infection of macrophages in vivo. (A) BALB/C mice were i.p. infected with 5×10^5^ PFU of wildtype or 131stopD MCMV. Six hours after infection PEC were harvested and CD11b^+^ F4/80^+^ macrophages analyzed for MCMV m06 expression by FACS analysis. The number of infected cells was significantly lower for the 131stop mutant. Shown are means +/−SD. Data were compiled from two independent experiments with altogether 9 mice analyzed per group. (B, C) Quantitation of infected F4/80^+^IE1^+^ liver-resident and recruited macrophages in situ. Two-color IHC analysis was performed on liver tissue sections derived from immunocompromised BALB/c mice on day 10 after intraplantar infection with viruses vpSM3fr, 131stopD, and wildtype virus. (B) Representative image of wildtype virus-infected liver tissue with red staining of the intranuclear viral protein IE1 for detecting infected cells and black staining of the marker antigen F4/80 (Ly71) for detecting macrophages. The red arrow exemplarily points to an enlarged hepatocyte nucleus containing the CMV-typical inclusion body that indicates productive infection. The black arrow exemplarily points to an uninfected F4/80^+^IE1^−^ macrophage. Black-and-red striped arrows highlight cases of infected F4/80^+^IE1^+^ macrophages. The bar marker represents 50 µm. (C) Quantitation of infected F4/80^+^IE1^+^ liver macrophages and statistical analysis. Symbols represent individual mice infected with the indicated viruses. Data represent numbers of F4/80^+^IE1^+^ macrophages counted per 10-mm^2^ areas of liver tissue sections and normalized to the total numbers of infected IE1^+^ cells and F4/80^+^ macrophages present in the same sections to take account of differences in the levels of overall infection and macrophage recruitment. Mean values and SDs are indicated. (A–C) P values were calculated by unpaired, two-tailed Student's t test with Welch's correction not assuming equal variance. Differences are considered significant for P<0.05.

When mice were infected with the 131stop mutant, a reduced virus production in salivary glands was observed (Fig. S4) as described for vpSM3fr [31] and for other MCK-2 mutants [28], [32]. To study macrophage infection in vivo, we infected adult BALB/c mice with wildtype and 131stopD MCMV and analyzed F4/80- and CD11b-double-positive macrophages from the peritoneal cavity 6 hours post infection. We observed a more than 50% reduction of the percentage of MCMV-infected macrophages when mice were infected with the 131stopD mutant (Fig. 5A). A significant reduction of normalized numbers of MCMV-infected macrophages could also be observed when immunocompromised BALB/c mice were infected via the footpad. Here, liver tissue sections were stained for F4/80 and MCMV IE1 protein 10 days after infection. Infected F4/80^+^IE1^+^ liver macrophages were counted and the numbers normalized to the numbers of all F4/80^+^ macrophages and all IE1^+^ cells present in the same tissue sections to take account of differences in the overall levels of infection and macrophage recruitment (Fig. 5B and C). Thus, very consistently, infection of immortalized macrophages, of primary bone marrow-derived macrophages, of macrophages ex vivo, and of macrophages in vivo were impaired when infected with MCMV lacking MCK-2.

### Both, MCMV gH/gL/gO and gH/gL/MCK-2 promote virus spread in cell culture

**Figure 6:**
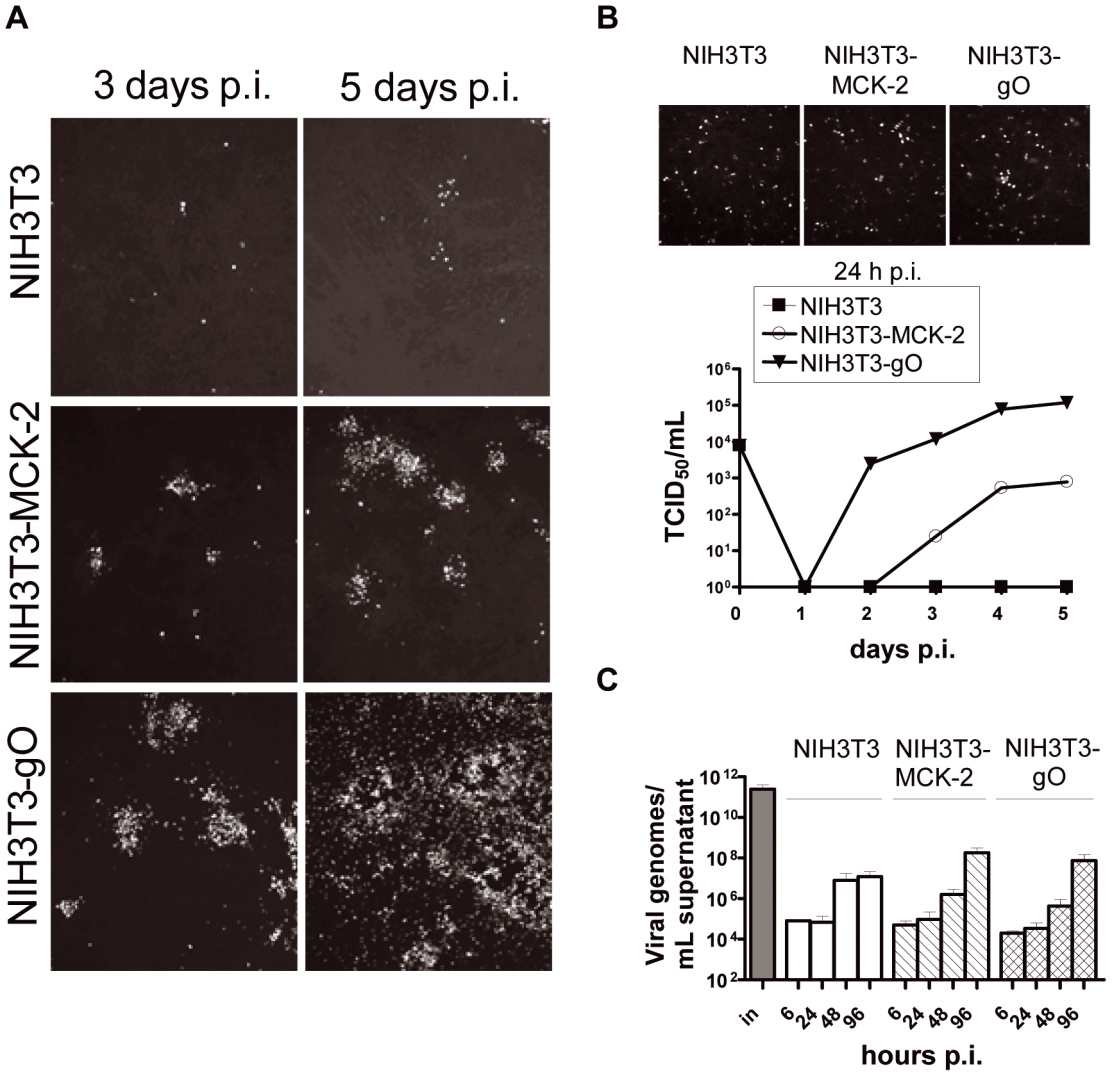
The patterns of gO- and MCK-2-dependent virus spread differ. NIH3T3, NIH3T3-MCK2, and NIH3T3-gO cells were infected at an m.o.i. of 0.1 with the double mutant Δm74/131stop reconstituted in NIH3T3-gO cells (A-C). (A) Spread in culture was followed by staining cells for IE1 expression 3 and 6 days after infection. (B) Infection was enhanced by a centrifugation step at 2,000× g for 30 min at RT. Cells were stained for IE1 24 h after infection to show the comparable initial infection of NIH3T3, NIH3T3-gO, and NIH3T3-MCK-2 cells (left panel). Release of infectious virus was monitored in multistep growth curves (right panel). (C) The release of DNAse-protected viral DNA was followed by real-time PCR using supernatants from the growth curves under (B, right panel) plus an additional time point at 6 h post infection. The column labeled “in” shows the amount of DNAse-protected viral DNA in the Δm74/131stop inoculum used to infect the cells. (A–C) p.i., post infection.

MCK-2 knock-out mutants only show very subtle phenotypes when their growth behavior is studied in vitro (Fig. 3 and 4). To evaluate the mechanism how MCK-2 controls infection of cells, it would be of advantage to analyze effects on a strong infection phenotype. For HCMV, it has been shown that knock-out of both, gO and pUL(128,130,131A) abolishes the capacity of the virus to infect cells [11]. Assuming that also MCMV either uses gH/gL/gO or gH/gL/MCK-2 for promoting entry into cells, knock-out of both proteins should also abolish its capacity to infect cells. To test this, we constructed double mutants lacking gO (Δm74) and additionally carrying stop cassettes either in the m129 ORF (129stop) or the m131 ORF (131stop). Reconstitution of both double mutants resulted in infected cells from which infection could barely spread (data not shown). Release of infectious virus into the supernatants could never be detected (data not shown). Yet, infectious supernatant virus carrying double mutations could readily be produced by virus reconstitution in NIH3T3 cells expressing gO (NIH3T3-gO) or MCK-2 (NIH3T3-MCK-2) (data not shown). Infection of these trans-complementing cell lines with the double mutants did not result in detectable levels of recombination between the mutated loci and the wildtype m74 or m131/129 ORFs of the trans-complementing cells (data not shown). We reconstituted the Δm74/131stop double mutant in NIH3T3-gO cells and used this virus to infect NIH3T3, NIH3T3-MCK2, or NIH3T3-gO cells. Double mutant virus produced in NIH3T3-gO cells should be gO-positive and MCK-2-negative and, after infection of new cells, virus progeny will be gO- and MCK-2-negative. If the new target cells are expressing gO then virus progeny will be gO-positive and MCK-2-negative. If the target cells are expressing MCK-2, virus progeny will be gO-negative and MCK-2-positive. Thus, we could study spread of a virus with an identical genetic backbone, but a different protein complementation. We either infected cells at a very low m.o.i. to study spread (Fig. 6A), or infection was enhanced by a centrifugation step to initially infect about 10% of cells to study virus production (Fig. 6B). Spread of the double mutant in NIH3T3 cells and, thus, in the absence of MCK-2 and gO was highly restricted (Fig. 6A, upper panel). Release of infectious virus, which was tested by titration of supernatants on NIH3T3-gO cells, could not be observed (Fig. 6B). Spread of the double mutant in cultures of NIH3T3-MCK-2 cells was predominantly focal (Fig. 6A, middle panel), and production of infectious supernatant virus was reduced when compared to the production by the double mutant growing in NIH3T3-gO cells (Fig. 6B). Thus, complementation of the MCK-2 defect of the double mutant resulted in a growth pattern comparable to the growth pattern observed for Δm74 or m74stop mutants [15]. In NIH3T3-gO cells the double mutant readily spread (Fig. 6A, lower panel) and produced infectious virus like wildtype virus (Fig. 6B). Thus, both, gO and MCK-2 could restore the spread deficiency of the double mutant, and for the first time we could show that MCK-2 is indeed promoting virus spread. MCK-2 restored not only efficient focal spread but also production of infectious virus. When supernatants where tested for DNAse-resistant viral DNA by real-time PCR, which should be an equivalent for DNA in virus particles, we found that independent of production of infectious virus, comparable numbers of viral DNA copies were released into the supernatants of NIH3T3, NIH3T3-MCK-2, and NIH3T3-gO cells (Fig. 6C). DNA copy numbers in the cell culture supernatants were identical at time points 6 and 24 hours after infection and reflect leftovers of the input supernatant (Fig. 6C). After 48 hours, the first round of replication was completed which was reflected by an increase in DNA copy numbers. In supernatants from infected NIH3T3 cells, the copy numbers were higher than in supernatants from trans-complementing cells. Very likely, this indicates that particles produced by NIH3T3 cells are not infectious, cannot enter new cells, and are accumulated, whereas particles from trans-complementing cells are infectious and infect new cells. At 96 h after infection, only supernatants from NIH3T3-gO and NIH3T3-MCK-2 cells which support efficient virus spread showed a further increase in DNA copy numbers mirroring a second round of infection (Fig. 6C). In summary, the experiments with the double mutant showed that even in the absence of gO and MCK-2, virus particles are produced and released, but they are not infectious. If the mutant is trans-complemented either with MCK-2 or gO, comparable numbers of virus particles are produced, but the infection capacities for fibroblasts seem to be lower when they are trans-complemented with MCK-2.

**Figure 7:**
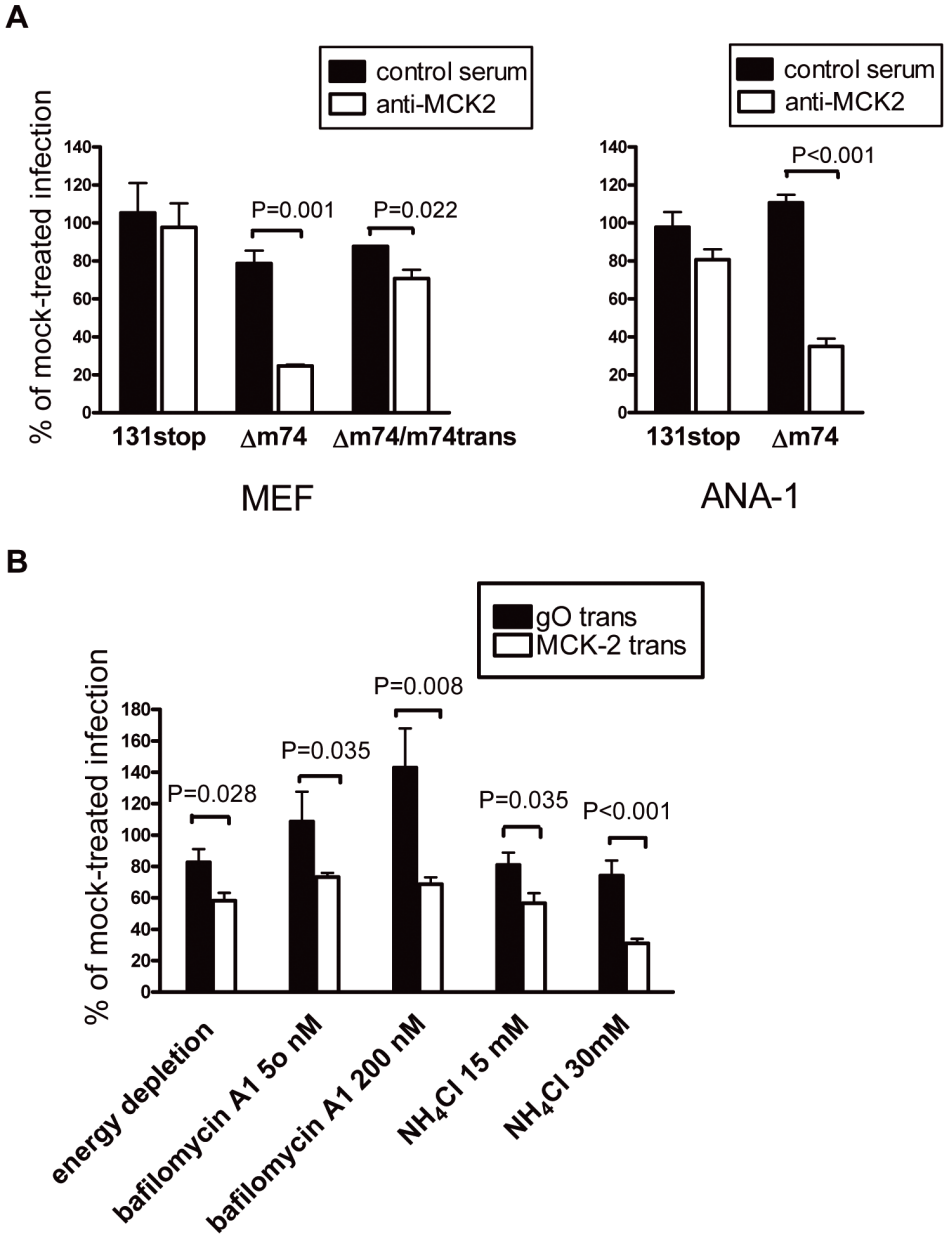
MCK-2 dependent infection of MEF. (A) MEF (left panel) and ANA-1 cells (right panel) were infected with MCMV mutants 131stopB and Δm74 and MEF additionally with Δm74/m74trans. The latter mutant was trans-complemented with gO by growth in NIH3T3-gO cells. Virus was either preincubated with anti-MCK2 rabbit antiserum or with an anti-pUL131A rabbit antiserum, which served as a control rabbit antiserum, at a dilution of 1∶10, or with medium as a mock control. Infection of cells was monitored by indirect immunofluorescence staining for IE1^+^ cells 6 h after infection. The percentage of IE1^+^ cells is expressed relative to the percentage of IE1^+^ cells of mock-treated infections. As indicated, both, Δm74 MCMV and Δm74/m74trans MCMV were significantly (Student's t-test) inhibited by the MCK-2 antiserum when compared to the control rabbit antiserum. The P values are indicated in the histograms. Shown are means +/−SEM of 3 to 4 independent experiments. (B) MEF were infected with a Δm74/131stop mutant either grown in NIH3T3-gO (gO-trans) or NIH3T3-MCK-2 (MCK-2 trans) cells in the presence or absence of energy depletion medium, bafilomycin A1, or NH_4_Cl. Three hours after infection, cells were fixed and stained for IE1 expression. The percentage of IE1^+^ cells is expressed relative to the percentage of IE1^+^ cells of mock-treated infections. For all inhibitors, inhibition of the gO trans-complemented and of the MCK-2 trans-complemented mutant was significantly different (Student's t test). The P values are indicated in the histograms. Shown are means +/− SEM of 4 to 6 independent experiments.

If gH/gL/MCK-2 is the alternative complex to gH/gL/gO with respect to promoting infection of host cells, antibodies directed against the MCK-2 complex should inhibit infection with MCMV lacking the gH/gL/gO complex but not infection with MCMV expressing gH/gL/gO. To study this, virus preparations were preincubated with a rabbit antiserum specific for MCK-2 or with a control rabbit antiserum. Then, cells were infected with these virus-antibody mixtures and infected cells were detected by staining the cells for expression of MCMV IE1. Numbers of infected cells were expressed as percent of infected cells obtained with mock-treated virus. Infection of MEF and ANA-1 cells with a Δm74 mutant (Fig. S1), could be strongly and specifically inhibited when virus was preincubated with the anti-MCK-2 antiserum, whereas infection with a 131stop mutant which expresses gO, but lacks MCK-2, could not be inhibited (Fig. 7A). Thus, in the absence of gO, infection is MCK-2-dependent. If MEF cells were infected with the Δm74 mutant trans-complemented in NIH3T3-gO cells, the inhibition by anti-MCK-2 antibodies was abrogated although not completely (Fig. 7A). This partial abrogation indicates that infection with the trans-complemented Δm74 mutant depends on gO and also on MCK-2.

We have shown before that infection of fibroblasts with MCMV lacking gO, but not with MCMV expressing gO, is energy- and pH-dependent [15]. To find out whether MCK-2 is promoting an energy- and pH-dependent entry pathway, we infected fibroblasts with a Δm74/m129stop mutant trans-complemented with gO or MCK-2 in the presence of inhibitors of ATP depletion or inhibitors of endosome acidification like bafilomycin A1 and NH_4_Cl [15]. Infection with MCK-2-complemented Δm74/m129stop MCMV was inhibited by all three inhibitors and inhibition was significantly different from inhibition of gO-complemented Δm74/m129stop MCMV (Fig. 7B). Bafilomyin A1 even increased infection of gO-complemented Δm74/m129stop MCMV. The inhibitor studies clearly indicate that MCK-2 promotes an energy- and pH-dependent entry pathway which is different from entry promoted in the presence of gO.

## Discussion

gH/gL complexes of herpesviruses have been extensively studied over the past years. The major function attributed to gH/gL associated proteins is receptor recognition. For HCMV, two gH/gL complexes, gH/gL/gO and gH/gL/pUL(128,130,131A) have been identified. gH/gL/gO determines entry into a restricted set of cell types and ensures efficient production of infectious supernatant virus in vitro [21]. The gH/gL/pUL(128,130,131A) complex determines the broad cell tropism characteristic for HCMV, very likely by recognizing a receptor found on many different cell types.

Infection of the mouse with MCMV has been shown to be a model for the HCMV infection in many, although not all, aspects [36]–[38]. We have recently characterized a functionally homologous gH/gL/gO complex of MCMV [15]. As MCMV mutants lacking gO can still infect cells and spread in cell culture, it was obvious that MCMV may also form a second gH/gL complex.

The role of the chemokine homolog MCK-2 of MCMV has been studied in vivo by using viruses in which the MCK-2 gene was deleted. Reduced salivary gland titers and reduced numbers of infected peripheral blood leukocytes have been attributed to the missing chemokine function of MCK-2. The observed phenotypes were explained by a role of MCK-2 in attracting myelomonocytic leukocytes to the site of infection which are then infected and promote dissemination and finally efficient infection of salivary glands [28]–[30], [32]. Recently, it has been shown that MCK-2 also attracts inflammatory monocytes which down-modulate antiviral CD8^+^ T cell responses [33]. Yet, these monocytes are not targets of infection. Additionally, it has been described that MCK-2 knock-out mutants not only recruit less myelomonocytic leukocytes to the site of infection but are also highly impaired in infecting them [30]. This pointed to an additional protein function of MCK-2 which drives infection efficiencies. However, this putative function has never been addressed.

Here, we propose a new function of MCK-2 which could explain the reduced infection efficiencies described above. We could show that MCK-2 forms a complex with gH which is incorporated into virions. It is known from crystal structures of other herpesviruses that gH and gL usually form tight heterodimers [2], [3], thus, the high molecular weight complex of gH and MCK-2 very likely is a gH/gL/MCK2 complex. In SDS-polyacrylamide gels, the complex showed a different size than the gH/gL/gO complex and anti-MCK-2 antibodies did not co-precipitate HA-tagged gO from extracts of cells infected with a virus expressing gO-HA indicating that the gH/gL/MCK-2 complex indeed is an alternative complex to gH/gL/gO.

It is difficult to study how MCK-2 promotes infection of cells in vitro, as spread and virus production of MCK-2 knock-out mutants are not drastically affected. Therefore, we used MCMV mutants lacking gO or double mutants lacking both, gO and MCK-2 to evaluate the contribution of MCK-2 to infection. Infection of cells with gO knock-out mutants could be blocked with anti-MCK-2 antibodies which demonstrated that gH/gL/MCK-2 can act as an alternative mediator of virus spread when gH/gL/gO is not formed. Trans-complementation of Δm74/131stop double mutants with MCK-2 showed that MCK-2 promotes mainly focal spread. Supernatants of cells infected with this virus only showed low titers of infectious virus, although high numbers of virus particles were released. This suggests that virions complemented with MCK-2, but lacking gO, are less efficient in infecting cells. In contrast to gO, MCK-2 promoted entry through a pH- and energy-dependent entry pathway as has been observed for MCMV mutants lacking gO. It is noteworthy in this context that in contrast to HCMV, where double mutants lacking gO and pUL(128,130,131A) are lethal [11], the MCMV double mutant can be reconstituted and spread in cell culture, although to a very limited degree and without producing free infectious virus. We do not know whether this residual spread occurs only by direct cell-to-cell transmission. It will have to be determined in the future whether MCK-2 and gO are directly involved in the entry process or whether they just promote infection as cofactors rendering target cells more susceptible for infection.

Whether gH/gL/MCK-2 is a tripartite complex or can associate with additional proteins is currently not known. Potential candidates would be the m130 and m133 genes which neighbor the m131/129 ORF. In an analysis of the MCMV transcriptome, we found that the putative m130 ORF which lies on the opposite strand and overlaps with m131/129 is not transcribed (data not shown). This is in line with data from Saederup et al. [32] who showed that interruption of the m130 ORF does not affect the phenotype of an m131/129 deletion mutant. It is intriguing that mutants lacking the m133 gene show, like MCK-2 mutants, reduced titers in salivary glands of infected mice [25], [26]. We could not detect peptides derived from the m133 ORF by mass spectrometry of anti-gH precipitates (data not shown). As this failure is not an absolute criterion to exclude that the m133 gene product is part of a gH/gL/MCK-2 complex, we deleted m133 and additionally m74. In contrast to MCK-2stop/Δm74 double mutants, the 133stop/Δm74 double mutant grew like a Δm74 mutant (data not shown).

We observed a slight growth advantage for MCK-2 mutants in fibroblasts with respect to production of supernatant virus which was not detected before [28], [31]. Interestingly, this finding is reminiscent of what was observed for UL131A mutants of HCMV [9], [39], and it might explain why isolates of MCMV do, just as isolates of HCMV, loose their capacity to form the second gH/gL complex during passage in fibroblasts [31], [40].

HCMV, which cannot form a gH/gL/pUL(128,130,131A) complex, completely loses its broad cell tropism in vitro, including its tropism for monocytes and macrophages, but can still infect fibroblasts like wildtype virus [41], [42]. This is a strong phenotype and it implies that for HCMV, infection of most cells types depends on the gH/gL/pUL(128,130,131A) complex. In contrast, deletion of MCK-2 was associated with a more restricted phenotype in vitro, namely, the loss of its capacity to efficiently infect macrophages. Additionally, an increased capacity to infect TCMK-1 epithelial cells was observed. This implies that the MCMV gH/gL/MCK-2 complex rather modulates infection capacities. Whether these differences reflect completely different roles for the gH/gL/MCK-2 complex of MCMV and the gH/gL/pUL(128,130,131A) complex of HCMV or are due to different in vitro culture systems is not known. Comparable to HCMV, rhesus CMV (RhCMV) lacking its gH/gL/pUL(128,130,131) complex shows reduced infection capacities for endothelial and epithelial cells [43], [44] but not for fibroblasts. Guinea pig CMV lacking its gH/gL/GP(129,131,133) complex loses its capacity to efficiently infect both, endothelial cells and fibroblasts [45].

Currently, it is not clear how these in vitro phenotypes translate to the in vivo infection. All CMV mutants, which lack the gH/gL complex containing a chemokine homolog, share one phenotype in vivo, namely, the loss of their capacity to efficiently establish infection in salivary glands [28], [32], [45]–[47]. We found that infection of mice with MCK-2 knock-out mutants results in reduced numbers of infected macrophages due to an impaired capacity of the mutants to infect the macrophages. How and whether this defect contributes to MCK-2 knock-out phenotypes like reduced viral titers in the salivary gland or elevated CD8^+^ T cell responses is currently not clear. It is also not known whether it is true for other cytomegaloviruses. Reduced virus replication of RhCMV mutants lacking gH/gL/pUL(128,130,131) is not restricted to salivary glands [47]. Yet, all in vivo studies performed so far used a RhCMV mutant which not only lacked a functional gH/gL/pUL(128,130,131) complex, but also additional viral genes coding for alpha chemokine-like proteins. When infection of different cell types in skin biopsies was tested for this RhCMV mutant a strong reduction in infection of endothelial cells and a slight, but not significant reduction in the numbers of infected macrophages was observed [48].

Both, MCMV gH/gL/MCK-2 and HCMV gH/gL/pUL(128,130,131A) contain potentially functional CC chemokines. Recombinant UL128 protein can interfere with the chemokine responsiveness of monocytes [49], and also isolated MCK-2 can act as a chemokine [29], [30]. The r129 gene product of rat CMV which is homologous to HCMV UL128 has also been shown to induce migration of lymphocytes as a recombinant protein [50]. At the moment it is not known whether MCK-2 and UL128 promote infection as gH/gL complex constituents and exert their chemokine functions only as free proteins or whether both functions can be complex-associated. Co-immunoprecipitation of gH and MCK-2 was only possible using an antibody recognizing the m131 ORF, but not the m129 derived protein part which indicates that the latter is involved in complex formation, whereas the part containing the CC chemokine domain is accessible. Thus, complex formation might still allow chemokine function of MCK-2. This also raises the question whether the chemokine function of MCK-2 and entry promoted by MCK-2 are both transmitted by the same cellular receptor. It will be of particular interest to find out whether it is possible to make MCK-2 mutants which are active chemokines, but no longer promote infection in the absence of gO or vice versa and to study them in vivo.

## Materials and Methods

### Cells and viruses

Primary mouse embryonal fibroblasts from BALB/c mice (MEF), NIH3T3 cells (ATCC: CRL-1658), the endothelial cell line MHEC5-T [51], the epithelial cell line TCMK-1 (ATCC: CCL-139), the macrophage cell line J774 (ATCC: TIB-67), and peritoneal exudates cells (PEC) from BALB/c mice were maintained in Dulbecco's modified Eagle's medium (DMEM) supplemented with 10% fetal calf serum. The macrophage cell line ANA-1 [52] was maintained in RPMI medium supplemented with 10% fetal calf serum.

BMDM were prepared from BALB/c mice. Femurs and tibias were removed and cleaned, and bone marrow was flushed through with DMEM supplemented with 10% FCS, 2 mM L-glutamine, 100 U/mL penicillin, 100 µg/mL streptomycin and 50 µM 2-mercaptoethanol. To remove stromal cells, bone marrow cell suspensions were first seeded in 10 cm tissue culture dishes for four hours. Then, non-adherent cells were collected, resuspended in complete medium additionally containing 20 ng/ml murine recombinant M-CSF (Peprotech), and cultivated for 7 days in 10 cm tissue culture dishes. During this time, non-adherent cells were removed daily and half of the medium was replaced by fresh, M-CSF containing medium. At day 7, cells were harvested and used for FACS analysis and subsequent experiments. More than 95% of the cells generated by this method stained positive for the macrophage marker F4/80 (data not shown).

As wildtype MCMV, a BAC-derived virus (pSM3fr-MCK-2fl) cloned from MCMV strain Smith was used [31]. pSM3fr BAC-derived virus was used as an additional m129 stop mutant [31], [53]. For infection experiments, supernatants from infected cells showing complete cytopathic effect (CPE) and precleared at 3,500× g were used. For production of supernatant virus for protein analysis, NIH3T3 cells were infected at an m.o.i. of 0.1. Media were collected when a full CPE was observed, cleared at 6,000× g for 10 min and then pelleted for 4 h at 20,000× g. Virus stocks for analysis of macrophage infection efficiencies were prepared as described recently [31]. Virus titers were determined by a TCID_50_ assay performed in 96 well plates on MEF or on NIH3T3-gO.

### Antibodies

Monoclonal mouse anti-MCK2 antibodies 5A5 and 2H9, rabbit anti-MCK2 antiserum WU1073 [27], and rabbit anti-pUL131A antiserum [9] have been described before. HA-tagged proteins were detected with rat anti-HA antibody (3F10, Roche Diagnostics). Mouse macrophages were stained with rat anti-F4/80 antibody (BM8, BioLegend). Mouse anti-MCMV gH (8D122A) was kindly provided by Lambert Loh, University of Saskatchewan, Canada. Mouse anti-MCMV immediate early protein 1 (IE1) antibody (Croma101) was kindly provided by Stipan Jonjic, University of Rijeka, Croatia.

### NIH3T3 cell lines expressing gO or MCK-2

An NIH3T3 cell line stably expressing gO has been described before [15]. For NIH3T3 cells stably expressing MCK-2, the complete m131/129 ORF was amplified by PCR from a pCR3-MCK-2 expression vector and cloned in a modified pEPi-luc vector [54] following the same strategy as used for pEPi-gO [15]. The resulting plasmid pEPi-MCK-2 was transfected into NIH3T3 cells using Fugene (Promega), and MCK-2 expressing cell clones isolated by limiting dilution under blasticidin S selection (10 µg/ml, Invivogen). MCK-2 expression was tested by staining cell extracts in the Western blot using an anti-MCK-2 antibody.

### Indirect immunofluorescence and intracellular FACS staining to monitor virus infection

For indirect immunofluorescence, adherent cells were fixed in 50% acetone-50% methanol and stained using anti-IE1 antibody and Fluor488-coupled goat anti-mouse antibody (Invitrogen). For counterstaining of cell nuclei, cells were incubated in PBS containing 5 µg/ml Hoechst 333258 (Invitrogen). For intracellular FACS staining, cells were detached with 0.5 mM Na-EDTA, fixed with 1% paraformaldehyde for 10 min and then stained in PBS containing 0.3% Saponin and 1% BSA using the antibodies described above. Cells were washed with PBS containing 0.03% Saponin. After staining, cells were resuspended in 1% paraformaldehyde and analyzed on a FACSCalibur using CellQuest software (BD Biosciences).

### Immunoprecipitation and Western blot analysis

Cells or virus pellets were lysed in RIPA buffer (50 mM Tris (pH 7.4), 150 mM NaCl, 1 mM EDTA, 1% NP-40, 0.1% SDS, 0.5% deoxycholate). Lysates were precleared with Sepharose G beads (GE Healthcare) and then, beads with antibody bound were added to the precleared lysates and coincubated for 4 h at 4°C. The beads were washed, proteins released in reducing sample buffer (0.13 M Tris-HCl (pH 6.8), 6% SDS, 10% α-thioglycerol) or in non-reducing sample buffer without α-thioglycerol and subjected to SDS-PAGE, followed by either Western blot analysis or LC-MS/MS.

### Liquid chromatography-tandem mass spectrometry (LC-MS/MS)

For preparation of peptides for LC-MS/MS, gel slices were chopped from the SDS-PAGE, treated with water and ammonium bicarbonate, and afterwards dehydrated using acetonitrile. Samples were reduced in DTT buffer (10 mM DTT, 40 mM ammonium bicarbonate) for 1 h and then alkylated with iodoacetamide buffer (55 mM iodoacetamide, 40 mM ammonium bicarbonate) for another 30 min in the dark. After washing in 40 mM ammonium bicarbonate, gel slices were dehydrated again in acetonitrile and soaked in 40 mM ammonium bicarbonate containing sequencing grade modified trypsin (Promega). Samples were incubated overnight at 37°C and resulting peptides were extracted by 5% formic acid, dried in a SpeedVac concentrator, resuspended in 15 µl 0.1% formic acid and analyzed in a nano-ESI-LC-MS/MS. Here, each sample was first separated on a C18 reversed phase column via a linear acetonitrile gradient (UltiMate 3000 system, Dionex) and column (75 µm i.d. ×15 cm, packed with C18 PepMap, 3 µm, 100 Å; LC Packings), before MS and MS/MS spectra were acquired on an Orbitrap mass spectrometer (Thermo Scientific). Recorded spectra were analyzed via Mascot Software (Matrix Science) using an MCMV protein database.

### BAC mutagenesis

Markerless BAC mutagenesis was performed to introduce stop cassettes in the m131/129 ORF, to delete 532 bp at the N-terminus of the m74 ORF, to introduce a C-terminal HA-tag to the M75 ORF and to introduce a C-terminal HA-tag to the m74 ORF in the pSM3fr-MCK-2fl BAC as described previously [11], [55]. A schematic presentation of the pSM3fr-MCK-2fl mutants is depicted in Figure S1. For the pSM3fr-m129stop BACmid (virus: 129stop), the primers m129stop-for (5′- GTACCGTTCCCGACCCAGGTGATCTCACAGACACACTCTATCCAGTTTTC**GGCTAGTTAACTAGCC**AGGATGACGACGATAAGTAGGG-3′) and m129stop-rev (5′-AATCGCCACGCATCACGGTGGGCAAGTACCCCTACGAGGTGAAGGACGGT**GGCTAGTTAACTAGCC**GAAAACTGGATAGAGTGTGTCAACCAATTAACCAATTCTGATTAG-3′) were used. For the pSM3fr-m131stop BACmid (virus: 131stop), the primers m131stop-for (5′-TGACCAGACACAAGAGTCTGTCCGACCACCAGGCCCGCTTAGCGCACACC**GGCTAGTTAACTAGCC**AGGATGACGACGATAAGTAGGG-3′) and m131stop-rev (5′-AACACTTCGTGCGGACGAGAGGTGGTTTTCACTACCTTCTCTGGGATGAG**GGCT** **AGTTAACTAGCC**GGTGTGCGCTAAGCGGGCCTCAACCAATTAACCAATTCTGATTAG-3′) were used. For the pSM3fr3-Δm74 BACmid (virus: Δm74), the primers deltam74-for (5′-TTTAAAATATTTGGCGGTGATGTTACTTTTCG GGGTGATGAGGTCTCTCCAGGATGACGACGATAAGTAGGG-3′) and deltam74-rev (5′-AGAGCCGCGATTAATGTCCGCTGTATTCAACGCGGAGATCAGCCCTCCC GGGAGAGACCTCATCACCCCGAAAAGTAACATCACCGCCAAATATTTTAAACAACCAATTAACCAATTCTGATTAG-3′) were used. For the pSM3fr-M75-HA BACmid (virus: gH-HA), the primers M75HA-for (5′-TAGCGATCCTCATGGCGCTAGGGCTGTACCGGC TGTGCCGGCAAAAAAGATACCCATACGACGTCCCAGACTACGCTAGGATGACG ACGATAAGTAGGG-3′) and M75HA-rev (5′-GACGCAATAAAGAATCTTTTCTTTCTTCATTCACCTCGCGTGTGTCCTTA**CTAAGCGTAGTCTGGGACGTCGTATGGGTA**CCGACACGGCCGTTTTTTCTCAACCAATTAACCAATTCTGATTAG-3′) were used. For the pSM3fr-m74-HA BACmid (virus: gO-HA), the primers m74HAfor (5′-AGAAACCACAACAACACGTACCGTCTCTGCCCCACAAAAGGCGCACCGGCTCAATATCCTTTAGCCGTGTC**TACCCATACGACGTCCCAGACTACGCT**AGGATGACGACGATAAGTAGGG-3′) and m74HA-rev (5′-GGCACTGGTGTTACAAGGCCTTCACCTCAGACACGGCTAAAGGATATTGA**CTAAGCGTAGTCTGGGACGTCGTA**TGGGTAGACACGGCTAAAGGATATTGAGCCGGTGCGCCTTTTGTGGGCAACCAATTAACCAATTCTGATTAG-3′) were used. This BAC also has a duplication of 18 C-terminal base pairs of m73 which overlapped with the C-terminus of m74. The sequences of the stop cassettes and the HA-tags in all primers are highlighted. Deletions and insertions of stop cassettes or HA-tags were controlled by restriction pattern analysis and subsequent sequencing. BACs were reconstituted to virus by transfection of BAC DNA into MEF using Superfect transfection reagent (Qiagen) according to the manufacturer's instructions. Transfected cells were propagated until viral plaques appeared, and supernatants from these cultures were used for further propagation.

### Density gradient purification of virus particles

Virus particles were purified from supernatants of MCMV-infected cells by Nycodenz-gradient purification [56]. Briefly, supernatants were cleared at 6,000× g for 10 min to remove cell debris, and then virions were pelleted by centrifugation at 20,000× g for 4 h. The resulting pellet was resuspended in VS-buffer (0.05 M Tris, 0.012 M KCl, 0.005 M EDTA (pH 7.8)) and free DNA removed by overnight treatment with 625 U/ml Benzonase (Novagen) at 4°C. Then, the suspension was loaded onto a continuous 10–40% Nycodenz (Axis-Shield) density gradient and separated at 20,000× g for 105 min at 4°C, and the band corresponding to virus particles was collected.

### Real-time PCR

100 µl supernatant from infected cells was pretreated with 75 U Benzonase for 20 min at RT to remove free DNA, and then DNA was extracted using the DNeasy blood and tissue kit (Qiagen). 1/20^th^ of the extracted DNA was used for real-time PCR which was performed on a Light Cycler (Roche Molecular Biochemicals) as described recently [57]. Primers used were specific for the MCMV M54 gene [15]. Viral DNA copy numbers/ml were calculated by comparing the amplification to standard curves using pSM3fr-LBR BAC DNA.

### Inhibition of virus entry

For energy depletion, cells were preincubated in energy depletion medium (glucose-free DMEM with 2% bovine serum albumin, 50 mM 2-deoxy-D-glucose, 0.1% sodium azide) for 1 h followed by coincubation with virus for 90 min in the presence of energy depletion medium. Virions that had not penetrated were inactivated by washing the cells two times with PBS pH 3.0. For inhibition of pH-dependent endocytosis, cells were pretreated with medium containing NH_4_Cl or bafilomycin A1 (Sigma) for 1 h at 37°C. Infection (90 min) and further incubations were all performed in the presence of the respective inhibitors. For all inhibitions, infection was monitored by staining cells for IE1 expression three hours after removing supernatant virus.

### Analysis of peritoneal macrophages after in vivo infection

Female BALB/c mice were housed and bred under specified-pathogen-free conditions at the Central Animal Facility of the Medical Faculty, University of Rijeka, in accordance with the guidelines contained in the *International Guiding Principles for Biomedical Research Involving Animals*. The approval of animal protocols has been obtained from the authorised Ethics Committee of the Croatian Ministry of Agriculture, Veterinary Department (Class: UP/I-322-01/13-01/31; No.: 525-10/0255-13-2). The animal care authorisation for the Central Animal Facility of the Medical Faculty, University of Rijeka has been issued by the Croatian Ministry of Agriculture, Veterinary Department (authorisation number: HR-POK-004). Eight- to 12-week-old mice were used in all experiments. The mice were infected intraperitoneally (i.p.) with 5×10^5^ PFU of wildtype or 131stopD in a volume of 500 µL. PEC collection: Mice were sacrificed 6 h p.i. and PEC were collected by washing the peritoneal cavity with 10 ml cold PBS. Erythrocytes were lysed, cells counted and 1×10^6^ cells stained for surface markers with the following antibodies: anti-F4/80-APC (BioLegend, BM8), anti-CD11c-PE (eBioscience, N418), anti-CD19-PerCP-Cy5.5 (eBioscience, eBio1D3), anti-CD11b-PECy7 (eBioscience, M1/70). Cells were then fixed using Cytofix/Cytoperm solution (BD) and Perm/Wash (BD) was used to dilute Abs for IC staining as well for washing. Cells were first incubated with CROMA229 (anti-m06) antibody and then with FITC-labeled rat anti-mouse IgG1 mAb (BD, A85-1). F4/80^+^CD11b^+^ macrophages were gated according to a recently published strategy [58] and analyzed for m06 expression. Flow cytometry was performed on FACSAria (BD Bioscience; San Jose, CA), and data were analyzed using the FlowJo software (Tree Star).

### Analysis of the in situ infection of liver macrophages

Female BALB/c mice were immunocompromised by total-body γ-irradiation with a dose of 6.5 Gy and infected in the left hind footpad with 10^5^ PFU of the indicated viruses. Mice were bred and housed under specified-pathogen-free conditions in the Central Laboratory Animal Facility (CLAF) at the University Medical Center of the Johannes Gutenberg-University, Mainz. Animal experiments were approved according to German federal law, permission numbers 23 177-07 and G10-1-052. Two-color immunohistochemical analysis (IHC) was performed on liver tissue sections at day 10 after infection. Macrophages were labeled specifically with a rat mAb directed against antigen F4/80 (Ly71; clone BM8, Acris antibodies). Black staining was achieved by using biotin-conjugated polyclonal anti-rat Ig (BD) and the peroxidase-coupled avidin-biotin complex (Vectastain Elite ABC kit, Vector Laboratories) with DAB as substrate and ammonium nickelsulfate hexahydrate for color enhancement. Infected cells were then labeled specifically with murine mAb CROMA 101, directed against viral protein IE1, and stained red with goat polyclonal alkaline phosphate-conjugated anti mouse IgG (AbD Serotec) and a fuchsin substrate-chromogen kit (Dako-Cytomation). Light blue counterstaining was performed with hematoxylin.

## Supporting Information

Figure S1 **Schematic presentation of the MCMV BAC mutants.** The positions of HA-tags, deletions and stop cassettes introduced in the wildtype (pSM3fr-MCK-2fl) genome are indicated. Only single mutants are depicted. The insert gives a more detailed picture of the m131/129 gene locus.
(TIF)

Figure S2 **Comparison of growth of wildtype and gH-HA MCMV in NIH3T3 cells.** Cells were infected at an m.o.i. of 0.5, supernatants harvested every 24 hours, titrated by a plaque assay and the titers expressed as plaque forming units (PFA) per ml. One representative growth curve is shown. p.i., post infection.
(TIF)

Figure S3 **Peritoneal exudate cells (PEC) were used as a source of macrophages.** PEC derived from untreated mice have been shown to consist of about 40 to 50% macrophages (F4/80^+^) [35]. PEC were stained with an anti-F4/80 antibody and analyzed by FACS. A) Cells in the macrophage gate (left panel) were 70 to 80% F4/80^+^ (right panel). These cells were used to analyze infection with wildtype and m131stop MCMV as shown in Fig. 4C (right panel). B) Less than 10% cells in the lymphocyte gate (left panel) were F4/80^+^ (right panel). For cells in this gate, we could not detect any MCMV infection (data not shown).
(TIF)

Figure S4 **131stop MCMV exhibits reduced titers in salivary glands after infection of mice.** BALB/c mice were i.p. infected with 2×10^5^ PFU of either vpSM3fr, 131stopD or wild type MCMV. On day 8 p.i., mice were sacrificed and viral titers in salivary glands were determined by plaque assay. Titers of individual mice (circles) and median values (horizontal bars) are shown. As indicated by asterisks, viral titers in salivary glands were significantly reduced after infection with vpSM3fr and 131stop MCMV when compared to wildtype infection (P<0.02, Student's t test). DL, detection limit.
(TIF)
