# Supplementary figures and images for: The Viral Chemokine MCK-2 of Murine Cytomegalovirus Promotes Infection as Part of a gH/gL/MCK-2 Complex
Source: PLoS Pathog. 2013 Jul 25;9(7):e1003493. doi: 10.1371/journal.ppat.1003493 (PMC3723581; doi:10.1371/journal.ppat.1003493)

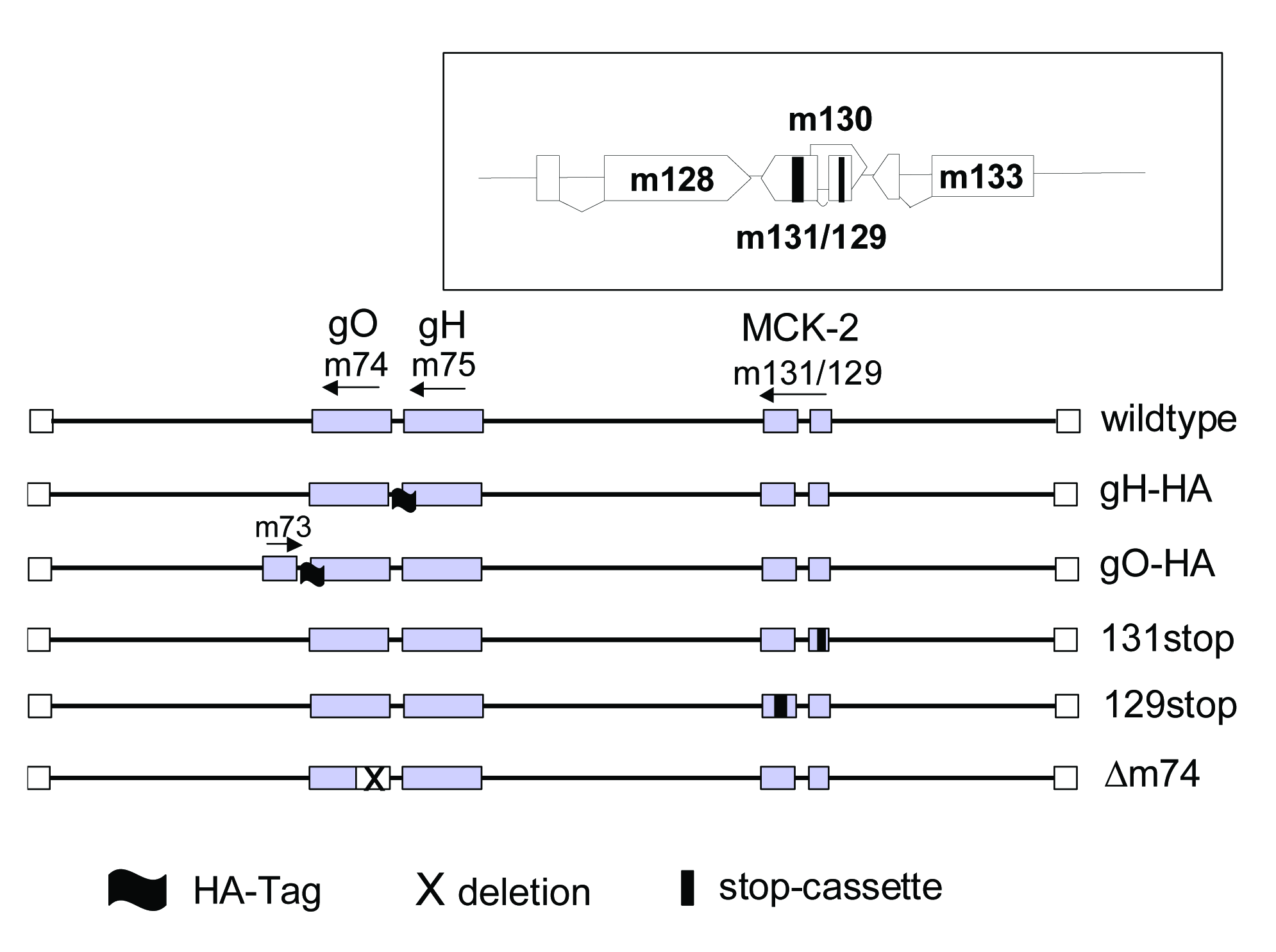

Supplement: Figure S1 — Schematic presentation of the MCMV BAC mutants. The positions of HA-tags, deletions and stop cassettes introduced in the wildtype (pSM3fr-MCK-2fl) genome are indicated. Only single mutants are depicted. The insert gives a more detailed picture of the m131/129 gene locus. (TIF) [file ppat.1003493.s001.tif]

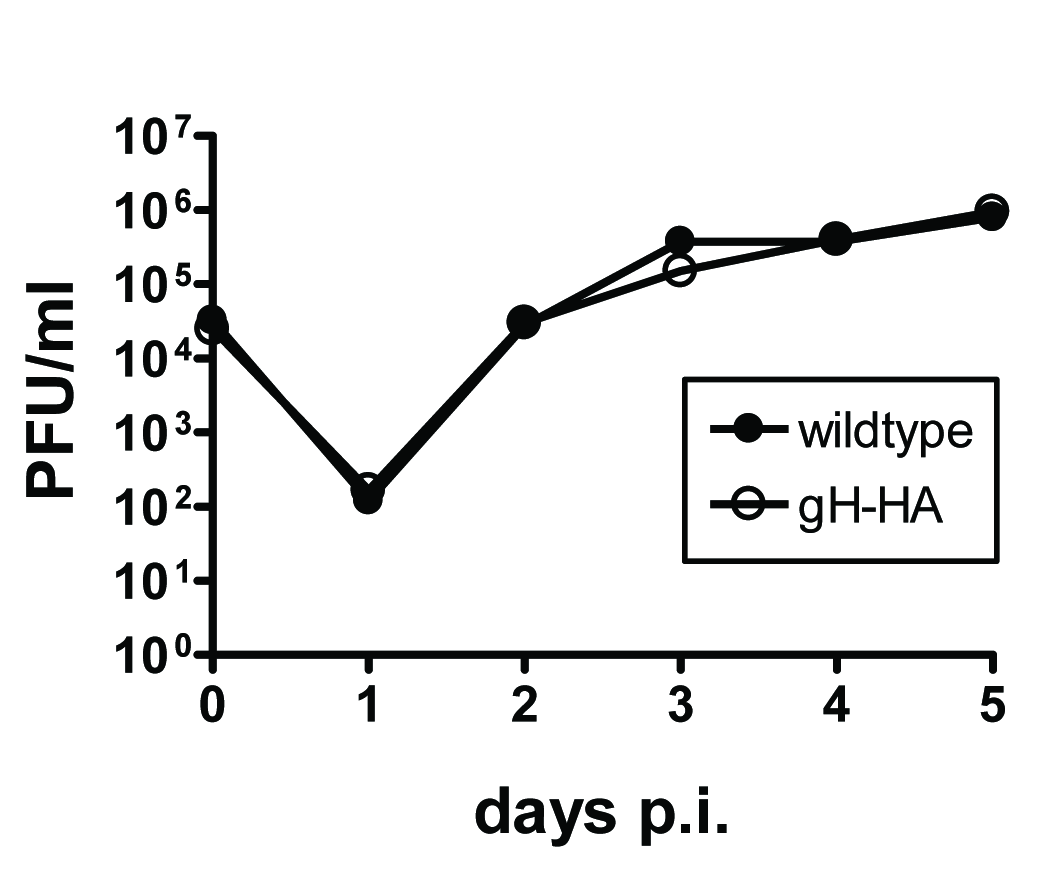

Supplement: Figure S2 — Comparison of growth of wildtype and gH-HA MCMV in NIH3T3 cells. Cells were infected at an m.o.i. of 0.5, supernatants harvested every 24 hours, titrated by a plaque assay and the titers expressed as plaque forming units (PFA) per ml. One representative growth curve is shown. p.i., post infection. (TIF) [file ppat.1003493.s002.tif]

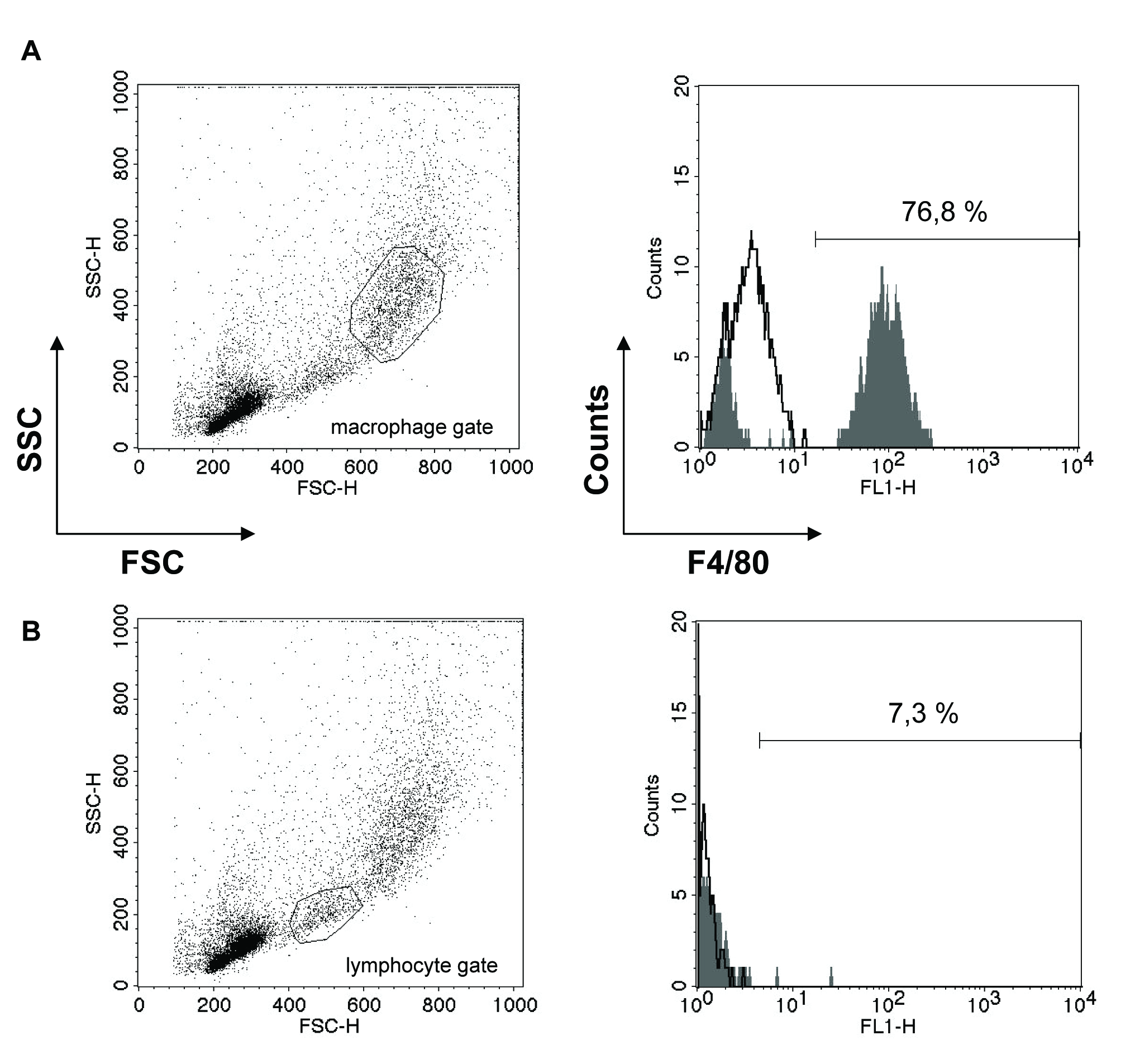

Supplement: Figure S3 — Peritoneal exudate cells (PEC) were used as a source of macrophages. PEC derived from untreated mice have been shown to consist of about 40 to 50% macrophages (F4/80+) [35]. PEC were stained with an anti-F4/80 antibody and analyzed by FACS. A) Cells in the macrophage gate (left panel) were 70 to 80% F4/80+ (right panel). These cells were used to analyze infection with wildtype and m131stop MCMV as shown in Fig. 4C (right panel). B) Less than 10% cells in the lymphocyte gate (left panel) were F4/80+ (right panel). For cells in this gate, we could not detect any MCMV infection (data not shown). (TIF) [file ppat.1003493.s003.tif]

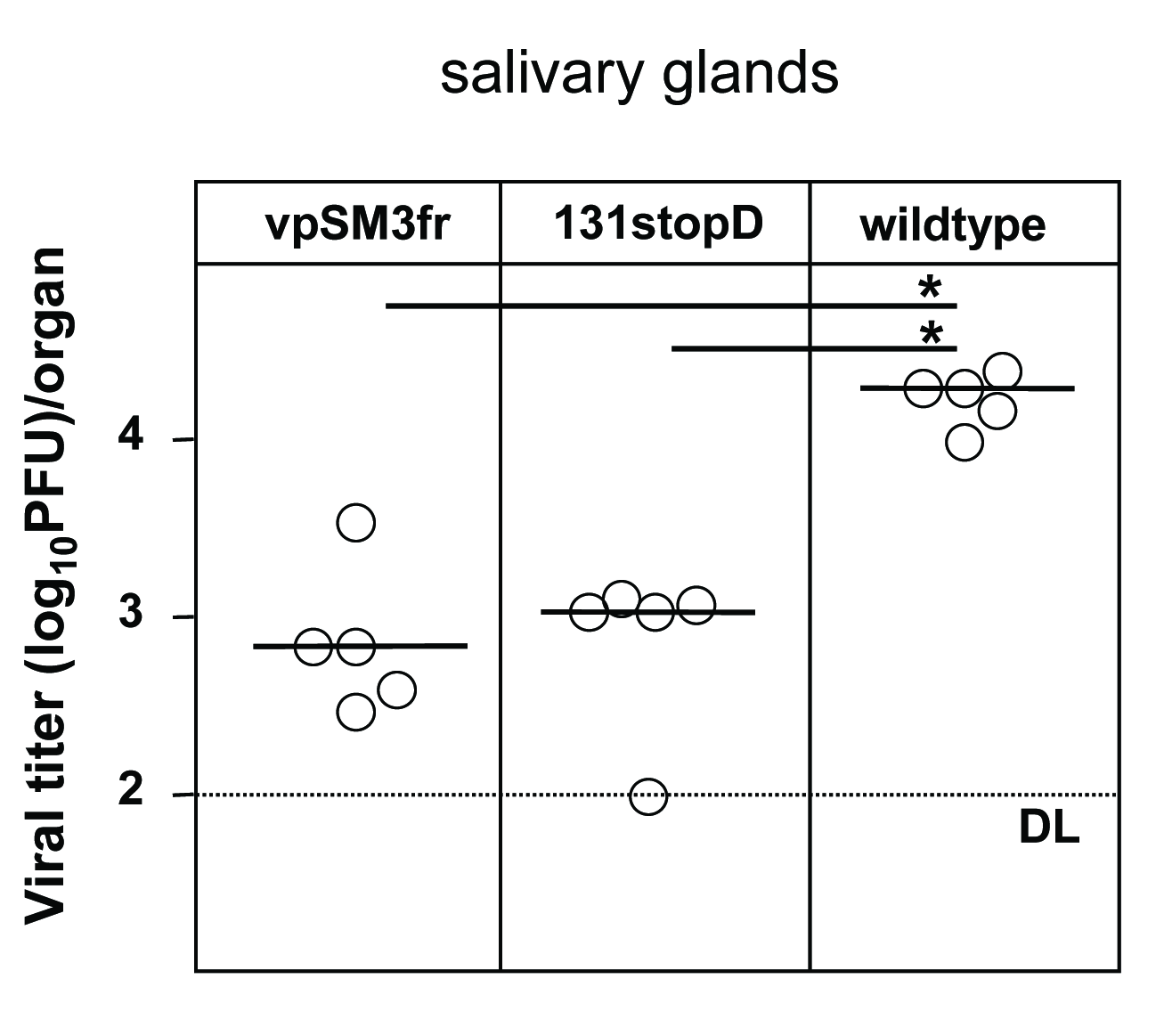

Supplement: Figure S4 — 131stop MCMV exhibits reduced titers in salivary glands after infection of mice. BALB/c mice were i.p. infected with 2×10^5^ PFU of either vpSM3fr, 131stopD or wild type MCMV. On day 8 p.i., mice were sacrificed and viral titers in salivary glands were determined by plaque assay. Titers of individual mice (circles) and median values (horizontal bars) are shown. As indicated by asterisks, viral titers in salivary glands were significantly reduced after infection with vpSM3fr and 131stop MCMV when compared to wildtype infection (P<0.02, Student's t test). DL, detection limit. (TIF) [file ppat.1003493.s004.tif]
